# Exacerbation of Chikungunya Virus Rheumatic Immunopathology by a High Fiber Diet and Butyrate

**DOI:** 10.3389/fimmu.2019.02736

**Published:** 2019-11-26

**Authors:** Natalie A. Prow, Thiago D. C. Hirata, Bing Tang, Thibaut Larcher, Pamela Mukhopadhyay, Tiago Lubiana Alves, Thuy T. Le, Joy Gardner, Yee Suan Poo, Eri Nakayama, Viviana P. Lutzky, Helder I. Nakaya, Andreas Suhrbier

**Affiliations:** ^1^Immunology Department, QIMR Berghofer Medical Research Institute, Brisbane, QLD, Australia; ^2^Australian Infectious Disease Research Centre, University of Queensland, Brisbane, QLD, Australia; ^3^Computational Systems Biology Laboratory, School of Pharmaceutical Sciences, University of São Paulo, São Paulo, Brazil; ^4^Institut National de Recherche Agronomique, Unité Mixte de Recherche 703, Oniris, Nantes, France; ^5^Department of Virology I, National Institute of Infectious Diseases, Tokyo, Japan

**Keywords:** chikungunya, immunopathology, arthritis, fiber, diet

## Abstract

Chikungunya virus (CHIKV) is a mosquito transmitted alphavirus associated with a robust systemic infection and an acute inflammatory rheumatic disease. A high fiber diet has been widely promoted for its ability to ameliorate inflammatory diseases. Fiber is fermented in the gut into short chain fatty acids such as acetate, propionate, and butyrate, which enter the circulation providing systemic anti-inflammatory activities. Herein we show that mice fed a high fiber diet show a clear exacerbation of CHIKV arthropathy, with increased edema and neutrophil infiltrates. RNA-Seq analyses illustrated that a high fiber diet, in this setting, promoted a range of pro-neutrophil responses including Th17/IL-17. Gene Set Enrichment Analyses demonstrated significant similarities with mouse models of inflammatory psoriasis and significant depression of macrophage resolution phase signatures in the CHIKV arthritic lesions from mice fed a high fiber diet. Supplementation of the drinking water with butyrate also increased edema after CHIKV infection. However, the mechanisms involved were different, with modulation of AP-1 and NF-κB responses identified, potentially implicating deoptimization of endothelial barrier repair. Thus, neither fiber nor short chain fatty acids provided benefits in this acute infectious disease setting, which is characterized by widespread viral cytopathic effects and a need for tissue repair.

## Introduction

A beneficial anti-inflammatory role for a high fiber diet is well-described for a large range of largely non-infectious disease settings in murine models (1–7). The use of high fiber diets to ameliorate human diseases is thus being actively pursued (8, 9), in particularly for autoimmune conditions (10, 11). Some evidence for benefit in humans has emerged, although results have often been inconclusive (12).

A high fiber diet changes the gut microbiome, with a number of studies in mice (3, 13, 14) and humans (15, 16) detailing the changes in bacterial species compositions. Bacterial fermentation of fiber (primarily undigested and/or indigestible carbohydrate) results in the production of short chain fatty acids (SCFA) such as acetate, propionate and butyrate, which enter the circulation and are believed to be the key players in dietary fiber-mediated systemic anti-inflammatory activities (8, 10, 17). SCFAs mediate effects on a number of cells including T cells (18, 19) particularly regulatory T cells (20), macrophages (21–23) and endothelial cells (24). Butyrate in particular has been shown to provide anti-inflammatory activities in a range of settings (5, 25–27), including non-infectious arthritic diseases (28–30). SCFAs are transported into cells via a series of receptors, with butyrate believed to act as an intracellular inhibitor of histone deacetylases (HDACs), with NF-κb (31), and AP-1 also targeted in some settings (32–34).

Chikungunya virus (CHIKV) belongs to a group of mosquito-borne arthritogenic alphaviruses that include the primarily Australian Ross River and Barmah Forest viruses, the African o'nyong-nyong virus, the Sindbis group of viruses, and the South American Mayaro virus (35). The largest documented outbreak of CHIKV disease ever recorded caused more than 10 million cases and began in 2004 in Africa and reached more than 100 countries in Africa, Asia, and the Americas, with small outbreaks also seen in Europe (36). Symptomatic infection of adults with CHIKV is nearly always associated with acute and often chronic polyarthralgia and/or polyarthritis, which can be debilitating and usually lasts weeks to months, occasionally longer. Other common symptoms include fever, rash, and myalgia (36). At present, no particularly effective drug or licensed vaccine is available for human use for any of these alphaviruses; although paracetamol/acetaminophen and non-steroidal anti-inflammatory drugs can provide relief from rheumatic symptoms and several CHIKV vaccines are in development (36).

CHIKV infection usually results in a 5–7 days long viremia, which is primarily controlled by a rapid type I IFN response (37, 38) and subsequently by anti-viral antibodies (36). A large range of cell types are infected *in vivo* including fibroblasts, muscle cells, endothelial cells, and macrophages (39). CHIKV infection usually results in cell death or cytopathic effects (CPE), mainly apoptosis and to a lesser extent necroptosis and pyroptosis, with connective tissue damage also evident during the viremic period in humans (36, 40). Infection drives a systemic pro-inflammatory response with the up-regulation of multiple mediators (36, 41, 42). CHIKV arthropathy is generally viewed as an immunopathology (43–45), with the pro-inflammatory arthritogenic response sharing similarities with rheumatoid arthritis (46). The inflammatory arthropathy is triggered by viral infection of joint tissues and is associated with a robust mononuclear cell infiltrate comprised primarily of monocytes, macrophages, NK cells, and T cells (47, 48). CD4 T cells are important for driving CHIKV arthritis (36), with Tregs associated with disease amelioration (49, 50). Macrophages/monocytes also play an important role in the arthritic immunopathology (36), with the pro-inflammatory response to CHIKV infection in peripheral blood shown to be monocyte centric (41, 51). However, macrophages are also required for resolution of inflammation, both generally (52–54) and specifically for CHIKV arthritic inflammation (45).

We have developed an adult C57BL/6J (wild-type) mouse model of acute and chronic CHIKV infection and hind foot arthritis that recapitulates many aspects of human disease (47, 55). RNA-Seq and bioinformatics studies in CHIKV patients (41) has also illustrated that this mouse model largely recapitulates (42) many of the inflammatory signatures seen in humans. CHIKV is able to replicate to high titers in humans with viremias up to 2.9 × 10^8^ pfu/ml (56) and even higher in the elderly (10^10^ viruses per ml of blood) (57). Similar titers are reached in the feet in the mouse model (47), with up to 8% of the polyadenylated RNA in the infected feet being of viral origin (42). The mouse model has been widely exploited for testing new interventions (43, 58–65), and is used herein to determine the potential for modulating CHIKV arthropathy with high fiber diet and SCFAs. Only a few studies (66, 67) have addressed the question of whether high fiber diet and/or SCFAs can provide anti-inflammatory benefits in infectious disease settings.

## Materials and Methods

### Mice and CHIKV Infection

C57BL/6J mice (6–8 weeks) were purchased from the Animal Resources Center (Canning Vale, WA, Australia). Female mice were inoculated with 10^4^ CCID_50_ of the Reunion Island isolate (LR2006-OPY1) in 40 μl of medium (RPMI1640 supplemented with 2% fetal calf serum), s.c. into both hind feet as described previously (47, 55). The virus (GenBank KT449801) was prepared in C6/36 cells (55). Serum viremia was determined by CCID_50_ assay using C6/36 and Vero cells as described (37, 55). Foot swelling was measured using digital calipers and is presented as a group average of the percentage increase in foot height times width for each foot compared with the same foot on day 0 (55).

### qRT PCR

qRT PCR was undertaken as described (55) using CHIKV E1 primers. Each sample was analyzed in duplicate and normalized to RPL13A mRNA levels.

### Diet and Water Supplementation

High and no fiber diets were supplied by Specialty Feeds (Glen Forrest, WA, Australia); the formulations are shown Supplementary Figures 1, 2 and were formulated to have similar digestible energy contents. Water for these later groups was acidified to pH 3–4 as per standard animal house practice. Drinking water was adjusted to pH 7 and supplemented with 200 mM of the sodium salt of the indicated SCFA (Sigma Aldrich, St Louis, MO, USA) (19, 68, 69). Drinking water was changed every 2–3 days. Mice were fed these diets and/or had SCFA supplementations in their drinking water for at least 3 weeks prior to CHIKV infection.

### RNA Isolation for RNA-Seq Analyses

C57BL/6 mice were infected with CHIKV as described above, and whole feet (cut above the ankle) harvested on day 6.5 post infection. Tissues were placed in RNAlater (Life Technologies, Carlsbad, CA, USA) overnight at 4°C and then homogenized into TRIzol (Invitrogen) and RNA extracted as described (42). There were four groups; high fiber and no fiber diets (water pH 3–4), and butyrate and water (pH 7) no fiber diets. For each group three biological replicates were created by pooling equal amounts of RNA from 3 to 4 feet from 3 to 4 mice. A total of 12 pooled RNA samples were DNase treated using RNAse-Free DNAse Set (Qiagen, Hilden, Germany), purified using an RNeasy MinElute Kit following the manufacturers' instructions.

### RNA-Seq Analyses

Library preparation and sequencing were conducted by the Australian Genome Research Facility (Melbourne, Australia). cDNA libraries were prepared using a TruSeq RNA Sample Prep Kit (v2) (Illumina Inc. San Diego, USA), which included isolation of poly-adenylated RNA using oligo-dt beads. cDNA libraries were sequenced from both ends (100 bp) using Illumina HiSeq 2000 Sequencer (Illumina Inc.). The CASAVA v1.8.2 pipeline was used to separate the bar-coded sequences and extract 100 base pair, paired end reads into FASTQ files.

### Differentially Expressed Genes

The read counts were used to determine gene expression and identify differentially expressed genes (DEGs) using R packages (R version 3.2.0) “edgeR” (3.18.1) and “limma” (3.32.7). (https://bioconductor.org/packages/release/bioc/html/edgeR.html). The default TMM normalization method of edgeR was used to normalize the counts. The GLM model was used to perform differential expression comparison between the groups. Genes that had >1 CPM in at least three samples were retained for further analysis. Differentially gene expression was considered significant if the Benjamini–Hochberg corrected *p*-value (i.e., FDR or q value) was <0.05. DEGs (*q* < 0.05) were analyzed by Ingenuity Pathway Analysis and Integrated System for Motif Activity Response Analysis (ISMARA) (70) as described (42, 71). ISMARA was undertaken by uploading the RNA-Seq fastq files, identifying the replicates (allowing averaging, *n* = 3) and undertaking pair-wise comparisons; high vs. no fiber (normal water) and butyrate vs. water (no fiber diet).

### Gene Set Enrichment Analyses (GSEAs)

#### Psoriasis Signatures

Enrichment analyses were performed using GSEA from *fgsea (v1.10.0)* R package (72), using up and down-regulated DEGs from the high fiber vs. no fiber diet comparison. The program *limma (v3.40.2)* R package (73) was used to determine the fold change (in all the genes) in datasets from murine models of inflammatory psoriasis models (GSE27628). These gene sets were ranked by log_2_ fold-change and considered as gene ranks in the GSEA.

Macrophage resolution phase signature. The microarray data (Gene Express accession number E-MEXP-3189) posted by Stables et al. was analyzed as described (52) to determine DEGs up-regulated in resolution phase macrophages (rM) when compared with naïve macrophages and inflammatory macrophages (Stables et al. provided a list of rM DEGs, but not the direction of expression change). The 146 up-regulated DEGs in rM were used in a GSEA with the 27,537 gene set (ranked by log_2_ fold-change) obtained by RNA-Seq analysis comparing feet day 7 post CHIKV infection with feet from mock infected mice (42). The up-regulated DEGs in rM were also used in a GSEA with the 34,624 gene set (ranked by log_2_ fold-change) obtained by RNA-Seq comparing CHIKV arthritis in mice fed a high and no fiber diet (feet day 6.5 post CHIKV infection) (Supplementary Table 1a).

### Histology and Immunohistochemistry

Histology, immunohistochemistry and quantitation were performed as described previously (45, 47, 71). Briefly, feet were fixed in paraformaldehyde, decalcified and embedded in paraffin, and sections stained with hematoxylin and eosin (H&E). Sections were scanned using Aperio AT Turbo (Aperio, Vista, CA) and analyzed using Aperio ImageScope software (v10) and the Positive Pixel Count v9 algorithm. Strong blue divided by total red pixels (default settings) represents a measure of cellular infiltration as leukocytes have a high nuclear to cytoplasmic area ratio.

For immunohistochemistry, sections were stained with rat anti-mouse Ly6G (catalog number NMP-R14; Abcam, Cambridge, MA, USA), with detection using Warp Red Chromogen Kit (Biocare Medical, Concord, CA, USA).

### Statistics

Statistics were performed using IBM SPSS Statistics (version 19). For mouse data the *t*-test was used if the difference in the variances was <4, skewness was >-2, and kurtosis was <2. Where the data was non-parametric and the distributions were similar the Kruskal–Wallis test was used, otherwise the Kolmogorov–Smirnov test was used. The Related Samples Wilcoxon Signed Rank test was used instead of a paired *t*-test as the paired data being compared was non-parametric.

## Results

### Fiber and Butyrate Exacerbate Peak Foot Swelling After CHIKV Infection

**Figure 1:**
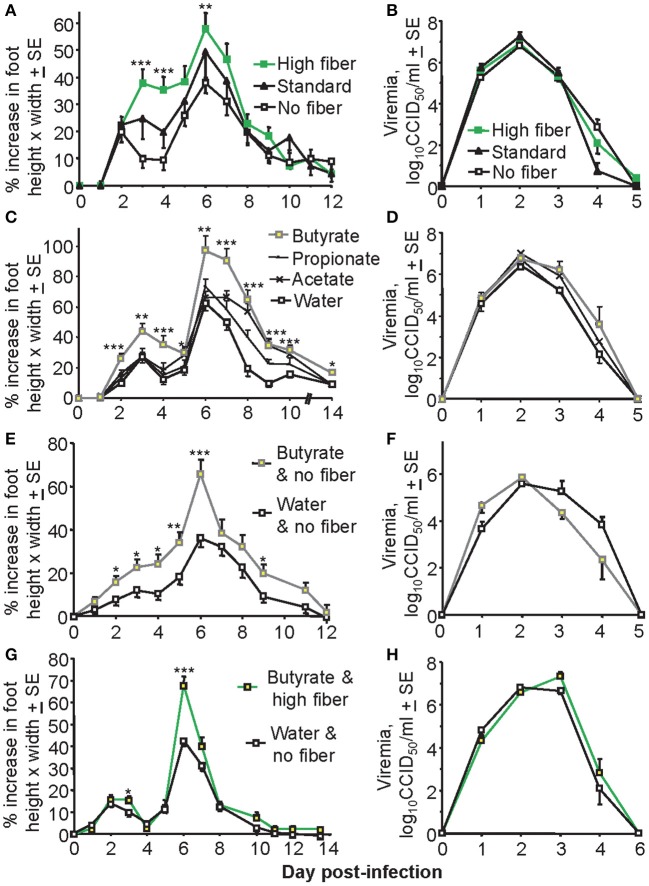
Diet affects foot swelling but not viremia. **(A)** Mice (C57BL/6J) were fed on either a high fiber diet, a standard diet or a no fiber diet for 3 weeks and were then infected with CHIKV and maintained on the same diet for the duration of the experiment. Foot swelling was measured over time and was higher in the high fiber group compared with the no fiber group; ***p < 0.001, **p = 0.008 (Day 7 approached significance *p* = 0.051), *t*-tests; *n* = 28–30 feet from 14 to 15 mice, with data from two independent experiments shown. **(B)** Viremia for the mice described in **(A)**. **(C)** Mice were fed on a standard diet and water was supplemented with 200 mM of the indicated SCFA for the duration of the experiment. Foot swelling was measured over time. Mice drinking butyrate had significantly increased foot swelling than control mice (standard water); ***p < 0.001, **p ≤ 0.003, *p < 0.04, *t*-tests; *n* = 12 feet from six mice. **(D)** Viremia for the mice described in **(C)**. **(E)** Mice were fed on a no fiber diet and the drinking water was supplemented with butyrate, or were fed a no fiber diet and supplied water for the duration of the experiment. After 3 weeks mice were infected with CHIKV and foot swelling measured. Butyrate significantly increased foot swelling; ***p < 0.001, **p = 0.01, *p < 0.04, *t*-tests; *n* = 22–24 feet from 11 to 12 mice, with data from two independent experiments shown. **(F)** Viremia for the mice treated as described in **(E)**; *n* = 6 mice, one experiment. **(G)** Mice were fed on a high fiber diet and the drinking water was supplemented with butyrate, or mice were fed on a no fiber diet and supplied unsupplemented water. After 3 weeks mice were infected with CHIKV and foot swelling measured. Butyrate/high fiber showed significantly increased foot swelling; ***p < 0.001, *p < 0.04, *t*-tests; *n* = 24 feet from 12 mice, with data from two independent experiments shown. **(H)** Viremia for mice treated as described in **(G)**; *n* = 6 mice, one experiment.

To investigate the effects of a high fiber diet on CHIKV arthritis, adult C57BL/6J mice were fed a high fiber, standard and no fiber diet for 3 weeks and were then infected with CHIKV as described (42, 47). Foot swelling was significantly higher in mice on a high fiber diet than in mice on a low fiber diet, with mice on a standard diet showing an intermediate phenotype (Figure 1A). Viremia in these groups of animals was not significantly different (Figure 1B).

Dietary fiber is fermented into SCFA, with supply of SCFA in drinking water frequently used to try and recapitulate the anti-inflammatory effects of a high fiber diet (28–30). Butyrate supplied in the drinking water to animals on a standard diet significantly increased foot swelling, with propionate and acetate being less active (Figure 1C). Again viremia was unaffected (Figure 1D). A similar effect on foot swelling was observed when mice on a no fiber diet were given butyrate to drink (Figure 1E), with viremia again not significantly affected (Figure 1F). When butyrate and a high fiber diet were combined no overt additive effects were observed (Figure 1G); the increase in mean maximum foot swelling (on day 6) mediated by butyrate was about 30% in Figures 1C,E and was also about 30% in Figure 1G. Viremia was again unaffected (Figure 1H).

Overall the most consistent statistically significant increase in foot swelling mediated by fiber or butyrate was on day 6, with days 6–7 usually representing the period of peak foot swelling that corresponds with a pronounced inflammatory infiltrate (45, 47).

### Viral Titers in Feet Were Unaffected by Fiber or Butyrate

**Figure 2:**
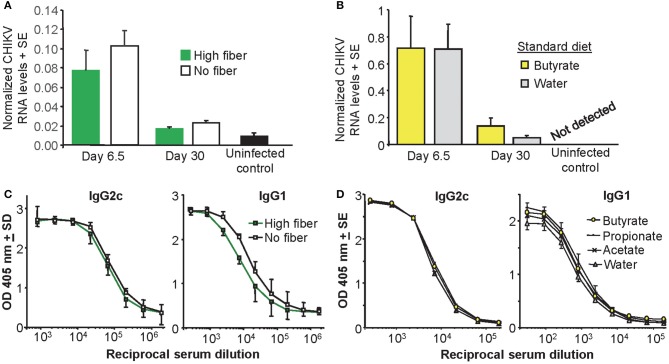
Viral loads and antibody responses. **(A)** Mice were fed on a high fiber or a no fiber diet for 3 weeks and were then infected with CHIKV (or left uninfected as controls). On day 6.5 and day 30 post infection feet were analyzed for CHIKV RNA by qRT PCR. Diet did not significantly affect CHIKV RNA levels (*n* = 4 mice and feet per group). **(B)** Mice were fed on a standard fiber and water was supplemented with butyrate or left unsupplemented (Water) and after 3 weeks mice were infected with CHIKV (or left uninfected as controls). Butyrate did not significant affect CHIKV RNA levels (*n* = 6 mice and feet per group). **(C)** Mice fed as for **(A)** with serum taken on day 30 post infection and assayed by ELISA for anti-CHIKV IgG2c or IgG1 antibody levels (*n* = 6 per group). **(D)** Mice with water supplementation as in **(B)**, with serum taken on day 30 post infection and assayed by ELISA for anti-CHIKV IgG2c or IgG1 antibody levels (*n* = 6 per group).

To determine whether the effects of fiber and butyrate on foot swelling were mediated by differences in viral loads in the foot tissues, CHIKV RNA levels were determined in feet by qRT PCR. CHIKV RNA levels were not significantly altered by a high fiber diet (Figure 2A) or butyrate in the drinking water (Figure 2B). This was true for both acute arthritis (day 6.5) and chronic arthritis, nominally measured on day 30 post infection (55) (Figures 2A,B). The differences in foot swelling seen in Figure 1 could thus not be accounted for by differences in viremia or viral loads in the feet.

### Anti-CHIKV Antibody Responses Are Similar After Fiber or Butyrate

Mice fed a high fiber diet showed no significant differences in their anti-CHIKV IgG2c or IgG1 responses post infection, when compared with mice fed a no fiber diet (Figure 2C). Although the IgG1 levels [associated with Th2 (74)] appeared a little lower (Figure 2C, IgG1), this did not reach significance when 50% end point titers in the two groups were compared. SCFA supplementation in the water supply also had no significant impact on anti-CHIKV IgG2c or IgG1 antibody titers (Figure 2D). These data failed to provide evidence of a significant effect of a high fiber diet or drinking butyrate on antibody responses and the Th1/Th2 balance in this CHIKV infection setting. These observations contrast with non-infectious disease settings where butyrate enhanced Th1 responses (75–77).

### High Fiber Diet Increases Edema and Neutrophil Infiltrates

**Figure 3:**
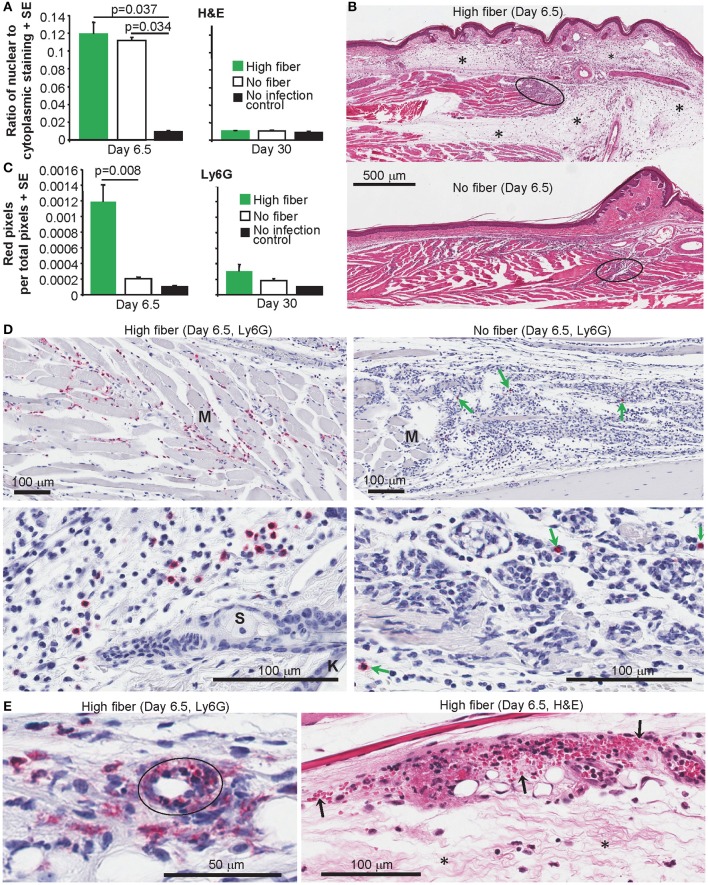
Histology and IHC of feet from CHIKV-infected mice fed high and no fiber diets. **(A)** Mice were fed with high fiber or no fiber diet and infected with CHIKV or left uninfected (No infection control) (as in Figure 1A). On day 6.5 and day 30 feet from separated groups of mice were examined by histology and H&E staining. Aperio pixel count was used to determine the ratio of strong blue (nuclear) to red (cytoplasmic) staining; a measure of the cellular infiltrate. Statistics by Kolmogorov Smirnov test (*p* = 0.037, *n* = 4 feet and mice per group) and Kruskal–Wallis test (*p* = 0.034, *n* = 3/4 feet and mice per group). There were no significant differences between any groups on day 30 post infection (*n* = 4 feet and mice per group). **(B)** Examples of H&E staining of mice examined in **(A)**. Black ovals indicate areas of high density infiltrates in muscle tissues. Asterisks (*) indicate areas of edema. **(C)** Feet from groups of mice as in **(A)** were analyzed by IHC and the neutrophil-specific anti-Ly6G antibody. Anti-Ly6G staining was detected using Warp Red, with red staining quantified by Aperio pixel count. Statistics by Kolmogorov Smirnov test (Day 6.5, *p* = 0.008, *n* = 4–9 feet and mice per group). There were no significant differences between any groups on day 30 post infection (*n* = 5/6 feet and mice per group). **(D)** Examples of anti-Ly6G IHC (Red) in muscle (top panels) and subcutaneous tissues (bottom panels). M, a muscle bundle; K, keratinocyte epidermal skin layer; S, Sebaceous gland. **(E)** Anti-Ly6G IHC (Red) showing a blood vessel (black oval) with intramural neutrophils and indications of parietal necrosis. H&E staining showing hemorrhage (extravascular red blood cells indicated by arrows) and edema (*).

CHIKV arthritis is characterized by a pronounced infiltrate of mononuclear cells into joint tissues (35, 45, 47, 78). The extent of the infiltrate can be quantified using the Aperio Positive Pixel Count Algorithm on digital scans of H&E stained foot sections, with infiltrating leukocytes having a higher blue (nuclear) to red (cytoplasmic) ratio (42, 55). As expected, substantial and significant infiltrates were evident after CHIKV infection; however, the high fiber and no fiber groups were not significantly different (Figure 3A). A clear increase in edema was, however, evident by H&E staining in the high fiber group (Figure 3B; other examples are shown in Supplementary Figure 3). This increase in edema likely accounts for the increased foot swelling seen in this group (Figure 1A). CHIKV-induced edema around peripheral joints is well-described in humans (79–82) and is recapitulated in this adult wild-type mouse model (47).

A characteristic feature of alphaviral arthritides is the general paucity of neutrophils in the arthritic lesions (45, 83, 84). In contrast, neutrophils are a prominent feature of autoimmune arthritides, such as rheumatoid arthritis (85). We have previously reported that in CCR2^−/−^ mice CHIKV arthritis is exacerbated by an increase in infiltrating neutrophils (45). As neutrophils can promote edema in various settings (86–89), foot sections from arthritic feet of CHIKV-infected mice fed high and no fiber diets were analyzed by immunohistochemistry (IHC) using anti-Ly6G staining [a neutrophil specific marker (45)]. Sections were digitally scanned and analyzed by Aperio Positive Pixel Count Algorithm. Significantly more anti-Ly6G staining was evident in the feet of mice (on day 6.5 post infection) fed a high fiber diet when compared to feet of mice fed a no fiber diet (Figure 3C, Day 6.5). No significant difference between groups was seen on day 30 post infection (Figure 3C, Day 30). Examples of the anti-Ly6G staining (red) from the high fiber group are shown for muscle (Figure 3D, top left), subcutaneous tissues (Figure 3D, bottom left) and synovium (Supplementary Figure 4). Fewer anti-Ly6G staining cells were observed in the no fiber group; this was clearly evident even when viewing areas containing high numbers of infiltrating cells (Figure 3D, green arrows, right hand panels).

Anti-Ly6G staining of feet of mice fed a high fiber diet also revealed occasional blood vessels with intramural neutrophils and indications of parietal necrosis (Figure 3E, Ly6G); such features were not seen in feet of mice fed a no fiber diet. Consistent with this observation were areas of hemorrhage in feet of mice fed a high fiber diet (Figure 3E, H&E), which were much less apparent in feet of mice fed a no fiber diet.

### The High Fiber Diet Promoted Pro-Neutrophil Responses

RNA-Seq was undertaken using mRNA from feet taken day 6.5 post CHIKV infection for mice fed high and no fiber diets. Quality assurance data for the RNA-Seq data are shown in Supplementary Figure 5. The full RNA-Seq gene counts are shown in in Supplementary Table 1a and differentially express genes (DEGs) with false discovery rate (FDR) <0.05 (q < 0.05) are listed in Supplementary Table 1b. This DEG list (with a *q* < 0.05 filter) was used for all subsequent bioinformatics treatments, unless stated otherwise. Amongst these 571 DEGs were a series of up-regulated cytokines/chemokines associated with neutrophil recruitment, survival and/or activation (Table 1; Supplementary Table 1b). The DEG list was analyzed using the Ingenuity Pathway Analysis (IPA) Upstream Regulator (USR) feature. The top USR by positive activation *z*-score was CEBPA (CCAAT/enhancer binding protein alpha) (Supplementary Table 1c), a transcription factor that induces neutrophilic differentiation (granulopoiesis) (90) (Table 1). The top USR by negative activation *z*-score was TP73 (p73) (Supplementary Table 1c), a p53-related protein, with p73^−/−^ mice showing massive neutrophil infiltrates and edema (112) (Table 1).

**Table 1:** Neutrophil signatures in DEGs and IPA USRs.

**DEG (*q* < 0.05)**	**Fold change**	**FDR**	**Activity**	
Cxcl2	2.42	3.1E-02	↑ neut. recruitment (91, 92)	
IL-6	1.93	4.9E-02	↑ neut. survival and migration (93, 94)	
IL-1β	1.71	4.0E-04	↑ neut. recruitment, survival, and activity (95–98)	
Cyr61	1.52	1.2E-03	↑ neut. infiltration (99)	
Ccr1	1.37	1.5E-02	↑ neut. migration/recruitment (100, 101)	
Ccl7	1.37	2.7E-02	↑ neut. chemotaxis (102)	
RORα	1.30	1.8E-02	↑ Th17 development (103)	
**IPA USR (direct only)**	**Activation** ***z*****-score**	***p*****-value**	**Activity**	
CEBPA	3.26	2.36E-05	↑ granulopoiesis (90, 104)	
HMGB1	1.755	2.00E-03	↑ neut. mediated injury (105)	
SMAD3	1.688	1.45E-04	↑ neut. activation (106)	
IRF-1	1.482	1.07E-02	↑ granulopoiesis (107)	
STAT4	1.136	1.04E-04	↑ neut. activation (108)	
EGR1	1.158	1.98E-04	Activated neuts. (109)	
HIF1A	1.052	2.74E-04	↑ neut. survival (110)	
DLX3	−2	3.39E-04	Dlx3^−/−^↑ skin IL-17 (111)	
TP73	−2.809	2.69E-04	p73^−/−^↑ neut. infiltration and edema (112)	
**IPA diseases or functions annotation (direct only)**			**Activation** ***z*****-score**	***p*****-value**
Accumulation of neutrophils			1.969	1.22E-03
Th17 immune response			1.698	2.18E-05
Polyarthritis			1.574	2.87E-06
Inflammation of joint			1.085	8.39E-04
Infiltration by neutrophils			0.528	2.78E-04
**IPA USR (direct and indirect)**	**Activation** ***z*****-score**	***p*****-value**	**Activity**	
IL-1β	2.962	4.84E-03	↑ neut. recruitment/survival/activity (95–98)	
PI3K/ERK	2.7/2.1	2.7E-03	↑ neut. SCFA-induced chemotaxis (113) and survival (114)	
		3.1E-04	
IL-17A	1.946	5.33E-06	↑ neut. recruitment and survival (114, 115)	
IL-10	−0.818	1.22E-03	↓ anti-inflammatory (116)	

*DEGs from RNA-Seq analysis of feet of CHIKV-infected mice (day 6.5 post infection) comparing high fiber diet with no fiber diets (Supplementary Table 1b), were analyzed by Ingenuity Pathway Analysis (IPA) upstream regulator (USR) feature (Supplementary Tables 1c,d). Selected DEGs and USRs associated with neutrophils (neuts.) are listed. FDR, false discovery rate (or q-value); ↑, increased; ↓, decreased*.

Rora (RORα) was present in the DEG list (Supplementary Table 1b), and represents a transcription factor this is associated *inter alia* with differentiation of Th17 cells (103) (Table 1).

The overall T cell infiltrate densities as measured by IHC for CD3 were not significantly different for high and no fiber groups (Supplementary Figure 6), arguing that Rora differences are not associated with differences in T cell recruitment. IL17A also identified as an USR when the IPA USR analysis was expanded to include both direct and indirect USR activities (Table 1, Supplementary Table 1e). IL-17 is a key cytokine that links T-cell activation to neutrophil mobilization and activation (117), with IL-17 playing an important role in promoting rheumatoid arthritis (115). The IPA USR analysis (direct and indirect) also returned (i) IL-1 and TNF, two cytokines known to increase endothelial permeability and edema (118–120) and (ii) PI3K and ERK, two kinases involved in survival and migration of neutrophils, including SCFA-induced chemotaxis (113) (Table 1, Supplementary Table 1e). These analyses support the IHC data (Figures 3C,D) and suggest that a range of inflammatory mediators and pathways that promote neutrophil-mediated inflammation were increased in mice fed a high fiber diet. Given the identification of IL17A as a USR (Table 1), the gene counts for the six IL-17 genes (IL-17a,b,c,d,f, and IL25) might be viewed as surprisingly low; although overall IL-17 counts were significantly higher for the high fiber group than the no fiber group (Supplemental Table 1a, see yellowed section, two way ANOVA, *p* = 0.028). The low counts may be due to an inherent feature of read alignment programs, which disregard reads that map to more than one gene. Although this process is important for accurate quantitation of transcripts, it results in loss of read counts for genes like IL-17 that exist as a family of homologous genes.

### Skin and Hair Genes, and Psoriasis Signatures After a High Fiber Diet

**Figure 4:**
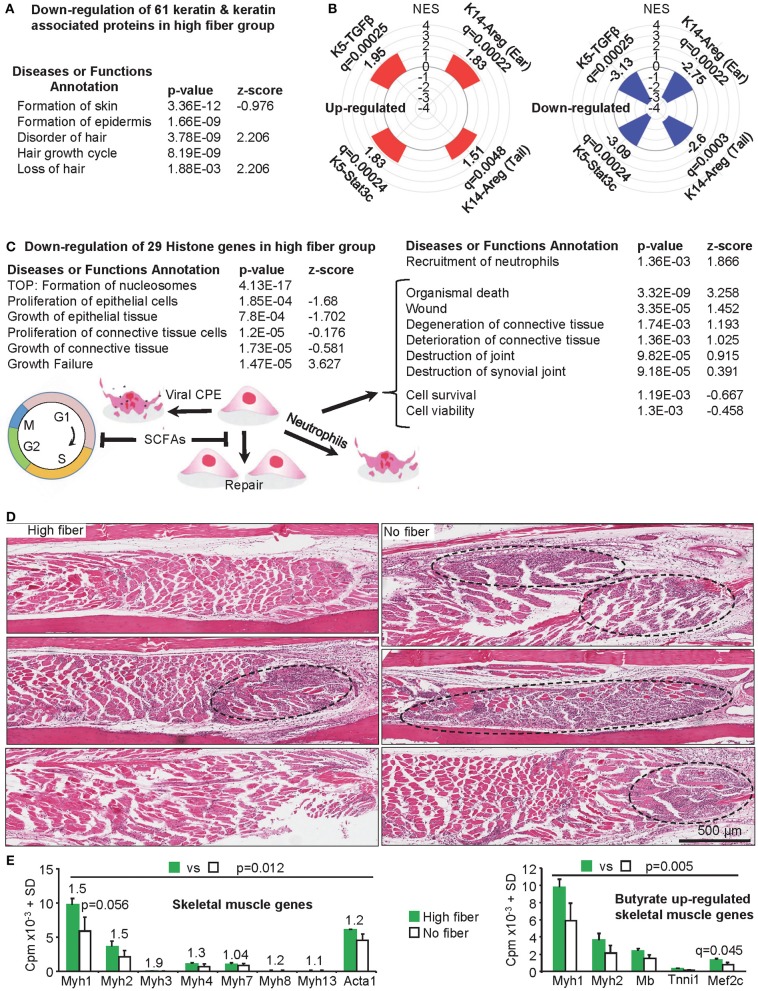
Additional changes mediated by a high fiber diet. **(A)** Skin and hair changes. The RNA-Seq data shows a significant down-regulation of 61 keratin and keratin associated proteins (Supplementary Table 1f). When the DEG list (Supplementary Table 1b, *q* < 0.05) was analyzed by IPA (direct only) using the *Diseases or Functions Annotation* feature, a range of annotations associated with skin and hair were identified (Supplementary Table 1d); the top four skin and hair annotations by *p*-value are shown. **(B)** Gene Set Enrichment Analyses was performed for up and down-regulated DEGs from high vs. no fiber diet (Supplementary Table 1b) and compared with gene sets pre-ranked by log_2_FC of inflammatory psoriasis mouse models (microarray study GSE27628): K14-Areg, over-expression of human amphiregulin in the basal epidermal layer (tail and skin); K5-Stat3c, basal keratinocyte-specific over-expression of a constitutively active mutant of signal transducer and activator of transcription 3; K5-TGFβ over-expression of the latent form of transforming growth factor β1 in basal keratinocyte. NES, Normalized Enrichment Score; q, FDR adjusted *p*-value. **(C)** Histone, proliferation and tissue destruction signatures. The RNA-Seq data shows a significant down-regulation of 29 histone genes in the high fiber group (Supplementary Table 1h). Formation of nucleosomes was the top *Diseases or Functions Annotation* by *p*-value (IPA analysis as in A), with a series of proliferation and growth annotations also returned with negative *z*-scores. The same analysis also returned a series of annotations associated with tissue destruction (right hand table) (Supplementary Table 1d). SCFAs (butyrate, propionate and valerate) can promote G1 cell cycle arrest and may thus inhibit proliferative replacement and repair of damaged tissues. CPE would contribute to tissue damage and neutrophils may also promote tissue destruction. **(D)** Skeletal muscle. H&E of skeletal muscle in feet of mice fed a high and no fiber diet day 6.5 post CHIKV infection are shown (three images from three mice per group). Muscle fibers (staining red/pink) appeared more extensively replaced by nuclear blue/purple staining of infiltrating inflammatory leukocytes in the no fiber group, with the latter also containing larger areas of dense blue/purple staining (dashed black ovals). **(E)** Left bar graph; mouse skeletal muscle myosin heavy chain and actin gene mean read counts (using read counts from Supplementary Table 1a). Numbers above the bars are fold change. No individual gene reached significance (*q* < 0.05); Myh1 approached significance by *p*-value. However, taken together significance was reached; statistics by Related Samples Wilcoxon Signed Rank Test. Right bar graph: skeletal muscle genes reported to be up-regulated by butyrate (121) were also up-regulated in the high fiber group, although only Mef2c reached significance. Taking the 5 genes together, differences between high fiber and no fiber reached significance; statistics by Related Samples Wilcoxon Signed Rank Test. For both graphs, SDs were derived from three biological replicates (Supplementary Table 1a).

The RNA-Seq analysis showed down-regulation of 61 keratin and keratin-associated-protein genes (Figure 4A, Supplementary Table 1f, *n* = 61) amongst the 402 down-regulated DEGs identified in feet during peak CHIKV-arthritis in mice fed a high fiber diet (Supplementary Table 1b). Most of these genes are expressed in skin, hair follicles and hair (Supplementary Table 1f). About half of these genes were previously shown to be down-regulated in the same setting in mice fed normal chow (42), perhaps consistent with the intermediate phenotype seen in Figure 1A.

Analyses of the down-regulated DEGs using Enrichr *Disease Perturbations from GEO*, suggested similarities with mouse models of psoriasis (Supplementary Table 1g). Gene Set Enrichment Analyses (GSEAs) were thus undertaken to compare both up and down-regulated DEGs from high vs. no fiber diet (Supplementary Table 1b) with publically available microarray data (GSE27628) from mouse models of inflammatory psoriasis (122). Genes up and down-regulated in CHIKV-infected mice fed a high fiber diet were also up and down-regulated in four mouse models of inflammatory psoriasis, respectively. All comparisons were highly significant by adjusted *p*-values (Figure 4B, Supplementary Table 1g). A high fiber diet thus increased skin inflammation after CHIKV infection with a signature similar to that seen in mouse models of inflammatory psoriasis.

Down-regulation of hair follicle genes (Supplementary Table 1f) is perhaps consistent with hair follicles being located next to areas of subcutaneous edema (Figure 3B, Supplementary Figure 7A) and adjacent to neutrophil infiltrates (Figure 3D, bottom left) (Supplementary Figure 7B). The presence of neutrophils in the epidermis or in the hair follicles, as has been described for psoriasis (123, 124), was not observed in our studies.

### Down-Regulation of Histone Genes and Increased Tissue Damage Signatures

Another prominent feature of the RNA-Seq data was down-regulation of 29 histone genes (mean fold change −1.75) in the high fiber group (Figure 4C, Supplementary Table 1h). Butyrate has been shown in several settings to promote cell-cycle arrest in G0/G1 via p21 and/or p15 (125), the former often via a p53-independent mechanism (126, 127). Such an activity would reduce transcription of many histone genes during S-phase (128). A p53-independent mechanism may also operate in the current setting, as overall p53 activity was decreased (Supplementary Table 1c, TP53). Two other SCFAs, propionate and valerate, can also promote cell-cycle arrest in G0/G1 in certain settings (129–133). The IPA *Diseases or Functions Annotations* showed multiple epithelial and connective tissue proliferation and growth annotations with negative z-scores (Figure 4C, Supplementary Table 1d), consistent with G1 arrest. CHIKV infections generate high levels of type I IFNs (37, 42, 71), which can also promote cell cycle blockage at G0/G1 (134); however, these cytokines were not differentially regulated in the high fiber vs. no fiber groups.

The IPA *Diseases or Functions Annotations* suggests that the high fiber diet promoted tissue damage during peak CHIKV arthritis (Figure 4C, right) (Supplementary Table 1d). Neutrophil infiltration may account for the latter, as we have previously shown that recruitment of neutrophils into joints in CHIKV-infected CCR2^−/−^ mice promotes cartilage damage (45), with neutrophils also associated with tissue damage in other settings (54). However, the overt histologically detectable cartilage damage seen in CCR2^−/−^ mice was not observed herein, with CCR2^−/−^ mice perhaps representing an extreme scenario given the complete absence of monocytes/macrophage infiltration during CHIKV arthritis (45). The tissue damage annotations (Figure 4C, right; Supplementary Table 1d) are unlikely to reflect differences in viral CPE, as viral loads were not different in the high fiber vs. no fiber groups (Figure 2B). However, given the high viral loads in feet (42, 47) extensive CPE would be expected in both groups, with reductions in cell proliferation in the high fiber group (Figure 4C, left) perhaps contributing to reduced proliferative tissue repair.

### Reduced Skeletal Muscle Damage in Mice Fed a High Fiber Diet

H&E staining of muscle tissues in the feet of CHIKV-infected mice fed a no fiber diet showed denser, more focal, inflammatory infiltrates in skeletal muscle tissues, when compared with CHIKV-infected mice fed a high fiber diet (Figure 4D, dashed black ovals). In these areas the red/pink staining of the muscle fibers was largely lost indicating more extensive muscle fiber degeneration in the no fiber group (Figure 4D). No compelling muscle signatures emerged from the bioinformatics analyses of the DEG list (Supplementary Table 1b). However, extracting mouse skeletal muscle myosin heavy chain (Myh1, 2, 3, 4, 7, 8, 13) and actin (Acta1) gene read counts from the full gene list (Supplementary Table 1a), illustrated that the mean read counts for these genes were always higher in the high fiber group; and taking all genes together, expression was significantly higher in the high vs. no fiber group (Figure 4E, left bar graph). Greater amounts of muscle specific mRNA species thus correlated with less muscle destruction in the high fiber group. Decreased skeletal muscle damage in the high fiber group, runs counter to the increased tissue damage or reduced tissue repair suggested by the analyses shown in Figure 4C. However, a recent report illustrated that butyrate supplementation promoted skeletal muscle formation in mice and up-regulated the expression of skeletal muscle genes, specifically, Myh1 and Myh2, myoglobin (Mb), and troponin-I, as well as the myocyte enhancer factor-2C (Mef2c), a key transcription factor for myogenesis (121). Extracting these genes from the full gene list (Supplementary Table 1a) illustrated that all these genes had higher mean read counts in the high fiber diet group, although only Mef2c reached significance (*q* = 0.045) (Figure 4E, right bar graph). When all five genes were taken together, their expression in the high fiber group was significantly higher (Figure 4E, right bar graph). Taken together, these results suggest that a high fiber diet is mildly myoprotective/myogenic in this CHIKV infection setting.

### The High Fiber Diet Modulates the Macrophage Resolution Phase Signature

Tissue injury usually results in inflammation and neutrophil recruitment, with subsequent resolution of inflammation and initiation of tissue repair requiring an active process that involves adoption of a resolution phase phenotype by macrophages. The resolution phase is usually characterized by apoptosis of neutrophils, the removal of apoptotic neutrophils by macrophages (efferocytosis), the prevention of further neutrophil recruitment and the initiation of tissue repair (53, 54, 135). In contrast to autoimmune diseases (85), the cellular infiltrates in alphaviral arthritides usually have few neutrophils (45, 83, 84), perhaps arguing that a resolution phase phenotype is present during peak arthritis. In this model viremia peaks days 1–3 and is usually over by day 5 post infection, with peak arthritis occurring days 6–7 post infection (47).

**Figure 5:**
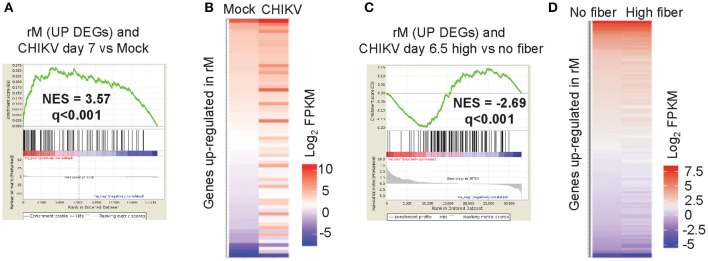
Macrophage resolution phase signature. The gene lists used in this figure are provided in Supplementary Table 1i. **(A)** A Gene Set Enrichment Analysis (GSEAs) comparing the 146 up-regulated DEGs associated with rM with the expression profile obtained from RNA-Seq analysis of feet day 7 post CHIKV infection vs. mock infected feet (18,517 genes pre-ranked by log_2_ expression). A normalized enrichment score (NES) and false discovery rate (*q*) is shown. **(B)** Heat map illustrating that the 69 core enriched genes identified by the GSEA (out of the 146 genes up-regulated in rM) are generally also up-regulated during peak CHIKV arthritis (day 7 post infection). **(C)** A GSEA comparing the up-regulated DEGs associated with rM with the expression profile obtained from RNA-Seq analysis of feet of mice fed a high fiber diet vs. feet of mice fed a no fiber diet day 6.5 post CHIKV infection (33,054 genes pre-ranked by log_2_ expression). **(D)** Heat map illustrating that the 126 core enriched genes identified by the GSEA (out of the 146 genes up-regulated in rM) are generally down-regulated during peak CHIKV arthritis in mice fed a high fiber diet.

To determine whether a resolution phase macrophage (rM) signature is present during peak CHIKV arthritis, a Gene Set Enrichment Analysis (GSEA) was undertaken to determine whether genes up-regulated in rM (52) were significantly represented during peak arthritis in feet of CHIKV-infected mice fed a normal diet (42). Using the microarray data posted by, and the methods described in, Stables et al. (52), we identified 146 genes that were up-regulated in rM (when compared with naïve macrophages and inflammatory macrophages) (Supplementary Table 1i). These DEGs were used in a GSEA using a pre-ranked (log_2_ fold change) list of the 18,517 genes obtained by RNA-Seq analysis of feet day 7 post CHIKV infection vs. mock infected feet (42) (Supplementary Table 1i). The GSEA provided a NES score of 3.57 and a high level of significance (*q* < 0.001), with 69/146 genes identified as core enriched genes (Figure 5A, Supplementary Table 1i). Genes up-regulated in rM were thus generally also up-regulated in arthritic feet day 7 post CHIKV infection, when compared with mock infected feet (Figure 5B). This analysis indicates that a significant rM signature is present in the feet of mice fed normal chow during peak CHIKV arthritis, perhaps explaining (at least in part) why there is a paucity of neutrophil infiltrates.

To determine whether the rM signature is modulated by a high fiber diet, a GSEA was undertaken using the same 146 genes up-regulated in rM and the pre-ranked 33,054 genes from CHIKV-infected feet from the high fiber vs. no fiber diet group (Supplementary Table 1i). The GSEA again providing a high level of significance, with 128/146 genes identified as core enriched genes (Figure 5C, Supplementary Table 1i). However, the NES score was negative (−2.69), with ≈35% of the genes up-regulated in rM down-regulated in feet of mice fed a high fiber diet (Figure 5D). Thus, the high fiber diet was associated with a significant down-modulation of the rM gene signature during peak CHIKV arthritis, which might explain (at least in part) the delayed clearance of neutrophils in the high fiber group (Figure 3C).

### Th2 Tissue Repair Signature

The type 2 immunity genes associated with tissue repair (IL-4, IL-13, IL-25, IL-5, TSLP, IL-33, TGFB1, and IL-10) (136) were not significantly lower in the high fiber group (Supplementary Table 1b). The IPA USR actually suggested an up-regulation of TGFB1 activity (Supplementary Table 1e, activation *z*-score 1.1, *p* = 0.0014), although this analysis did show down-regulation of IL-10 (Table 1). The first five genes in the aforementioned list also showed very low expression levels (Supplementary Table 1a), consistent with the dominant Th1 bias associated with CHIKV infection in this model (45, 47). That fiber has no significant effect on Th1/Th2 is supported by Figure 2C.

### CHIKV Rheumatic Disease After Drinking Butyrate

**Figure 6:**
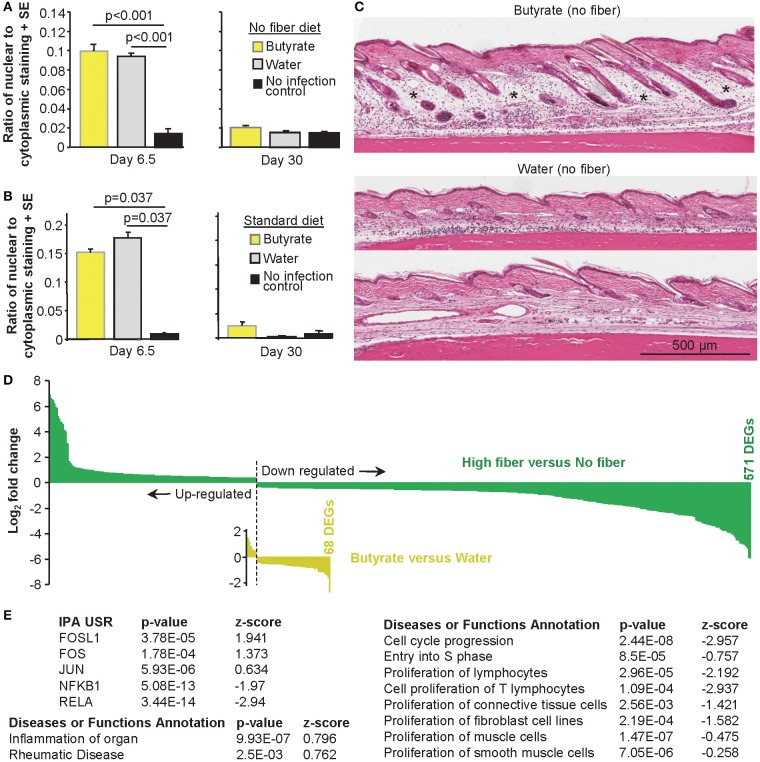
The effects of butyrate on CHIKV rheumatic disease. **(A)** Mice were fed a no fiber diet and given water supplemented with and without butyrate as in Figure 1E. On the indicated day, feet were examined by H&E and Aperio pixel count as in Figure 3A. Statistics by *t*-tests, *n* = 6 mice and feet per group. **(B)** As for **(A)**, but mice were fed on a standard diet as in Figure 1C. Statistics by Kolmogorov–Smirnov tests *n* = 3–6 feet and mice per group. **(C)** H&E images of foot sections of mice treated as in **(A)**. *Edema. **(D)** Bar graph of fold change of all 571 DEGs (*q* < 0.05) for high fiber vs. no fiber (green) and 68 DEGs for butyrate vs. water (yellow). Full data sets available in Supplementary Tables 1b,k. **(E)** IPA USR and *Diseases or Functions Annotation* analyses (direct only) of the 68 butyrate DEGs. Full data sets available in Supplementary Tables 1l,n.

The effects of high fiber diet are often associated with butyrate, with butyrate substitution in the drinking water (like high fiber diet) also able to exacerbate CHIKV-induced foot swelling significantly (Figures 1C,E,G). As observed after a high fiber diet (Figure 3A), drinking butyrate did not significantly increase the levels of cellular infiltrates in feet 6.5 days after CHIKV infection compared to drinking unsupplemented water (Figure 6A). This remained true when mice drank butyrate and were fed a standard diet (Figure 6B). Similar to the observations made for the high fiber diet group (Figure 3B), increased edema was again discernible by H&E in the feet of CHIKV-infected mice drinking butyrate (Figure 6C).

IHC staining for neutrophils illustrated very low levels of neutrophils in both butyrate and water (no fiber) groups, with marginally more neutrophils in the butyrate group, but this was not significant (Supplementary Figure 8).

### RNA-Seq of Butyrate vs. Water

RNA-Seq was undertaken using mRNA from feet of mice harvested on day 6.5 post CHIKV infection after mice were given either butyrate or unsupplemented water to drink; both groups were on a no fiber diet (drink and diet conditions also used for Figures 1E, 6A). Quality assurance data for the RNA-Seq are shown in Supplementary Figure 5. The full RNA-Seq gene counts are provided in Supplementary Table 1j and differentially express genes (DEGs) with false discovery rate (FDR) *q* < 0.05 are listed in Supplementary Table 1k and were used for all subsequent bioinformatics treatments, unless stated otherwise.

The number of DEGs for butyrate vs. water was only 68 (compared with 571 for high vs. no fiber diet, *q* < 0.05 for both), illustrating that the perturbations in CHIKV rheumatic disease mediated by drinking butyrate were substantially less widespread than those engendered by a high fiber diet (Figure 6D). This observation is perhaps consistent with the more complex role played by fiber, which *inter alia* produces a range of different SCFAs (18, 137). In addition, drinking butyrate resulted in only two genes with a fold change (FC) >2.8 (log_2_ of 1.5) and a maximum fold change of 6.5 (log_2_ of 2.71). In contrast, a high fiber diet resulted in 144 genes with FC>2.8 and a maximum fold change 118 (log_2_ of 6.88). The overall magnitude of transcriptional perturbations were thus also substantially lower for butyrate (Figure 6D).

There was no overlap in DEGs identified for “butyrate vs. water” and “high vs. no fiber” when a FDR *q* < 0.05 filter was applied to both. When the significance filter for “butyrate vs. water” was reduced to *p* < 0.05 (i.e., a *p*-value filter without FDR adjustment), only a small overlap of six genes for up-regulated genes (with 169 DEGs for high fiber *q* < 0.05 and 692 genes for butyrate *p* < 0.05) and 46 genes for down-regulated genes (402 DEGs for high fiber *q* < 0.05 and 1,220 genes for butyrate *p* < 0.05) became apparent. IPA USR analyses illustrated an overlap of (i) 2 USRs (FOS and JUN) for the 9 USRs (for butyrate vs. water) and the 34 USRs (for high vs. no fiber) with positive *z*-scores, and (ii) an overlap of 8 USRs (NOTCH1, CEBPB, STAT3, FOXO3, TP53, CREM, CTNNB1, KLF4, and PPARG) for the 45 USRs (for butyrate vs. water) and the 29 USRs (for high vs. no fiber) with negative *z*-score (Supplementary Tables 1c,l).

The “Integrated System for Motif Activity Response Analysis” (ISMARA) (70) provides an independent analysis of the RNA-Seq data and generates an activity score for usage of known transcription factor binding sites as determined from the promoter regions of genes identified by RNA-Seq. ISMARA provides a *Z*-value, with higher *Z*-values suggesting more significant differences between the two groups. ISMARA identified differential use (with *Z* > 1.5) of 11 transcription factor binding sites for “butyrate vs. water” and 60 for “high vs. no fiber.” Three of these sites were shared and showed the same direction of change (Supplementary Table 1m). Thus, although both butyrate and high fiber increased peak foot swelling and promoted edema after CHIKV infection, the mechanisms involved appear to be largely different.

### Butyrate Modulates AP-1 and NF-κB During CHIKV Arthropathy

Analysis of the 68 DEGs (Butyrate vs. Water; Supplementary Table 1k) using the USR feature of IPA (direct only), indicated that butyrate consumption up-regulated (positive *z*-score) pathways associated with FOSL1 (FRA-1), FOS (c-FOS), and JUN during peak CHIKV arthritis (Figure 6E, Supplementary Table 1l). c-JUN, JUNB and JUND, and c-FOS, FOSB, FRA-1 and FRA2 combine to form AP-1 transcription factor complexes, that can also include Maf and ATF family members (138). Modulation of AP-1 complexes was further supported by down-regulation of JUNB, FOSB, and FOS amongst the 68 DEGs (Supplementary Table 1k), with ATF4 down-regulation also identified by the IPA USR (Supplementary Table 1l). ISMARA also suggested modulation of AP-1 activities, although only Junb-Jund reached, and Fosl2 approached, significance (Supplementary Figure 9, Supplementary Table 1m). Butyrate has been shown to modulate AP-1 activities in several settings (32–34), with AP-1 modulation previously associated with edema (139, 140) and inflammation (141). Both FOS and JUN were also identified (with positive *z*-scores) in the IPA USR analysis of high vs. no fiber DEGs (Supplementary Table 1c).

The IPA USR analysis suggested that several NF-κB, STAT, and IRF pathways were down-regulated during peak CHIKV arthritis in mice drinking butyrate (Supplementary Table 1l). NF-κB inhibition was quite marked and highly significant (Figure 6E), consistent with this pathway being a major target of butyrate activity (31, 142). NFKBIA (NF-κB inhibitor alpha) was also identified (with positive *z*-score) in the IPA USR analysis of high vs. no fiber DEGs (Supplementary Table 1c).

Using the *Diseases or Functions Annotation* feature of IPA, significant annotations for inflammation and rheumatic disease were returned (Figure 6E), consistent with Figures 1E, 6C. However, the dominant signature (with multiple annotations; Supplementary Table 1n) was cell cycle arrest, reduced growth and/or reduced proliferation (Figure 6E, right hand table); an observation consistent with the ability of butyrate to promote cell cycle arrest (125–127). However, the lack of histones in the DEG list (Supplementary Table 1k) suggests this effect was less substantial than that seen after a high fiber diet, consistent with Figure 6D.

### Anti-Viral Activity Was Unaffected by Fiber or Butyrate

The IPA USR analysis (direct only) suggested the activities of several transcription factors associated with type I IFN responses (42, 71) are changed in the arthritic feet of mice fed high fiber diet, although activation *z*-scores were generally low (e.g., IRF8, STAT1, STAT3, NF-κB family, CTNNB1, ATF3; Supplementary Table 1c). No USRs central to the anti-CHIKV innate responses were identified [e.g., IRF3, IRF7, IFNAR (37)] and the DEG list (Supplementary Table 1b) had few, if any, effectors known to mediate anti-viral activity against CHIKV (42). These results are consistent with the lack of significant changes in viremia (Figure 1B) or viral loads in feet (Figure 2A). In addition, RNA-Seq reads can be mapped to the CHIKV genome (42), with the number of reads similar for high fiber verse no fiber groups (Supplementary Figure 10).

Butyrate substitution in the drinking water also did not affect viremia (Figure 1F) or viral loads in feet (Figure 2B) or RNA-Seq reads mapping to the CHIKV genome (Supplementary Figure 10). However, a number of innate genes/activities associated with antiviral activity against CHIKV (42) were down-regulated by butyrate (i) Mx1 (143), ≈1.5 fold down-regulated (Supplementary Table 1k), (ii) IRF3 and STAT1 activities by IPA USR analyses (Supplementary Table 1l, *z*-score −1.85 and −1.33, respectively), and (iii) Irf2_Irf1_Irf8_Irf9_Irf7 site usage (Supplementary Figure 9A, down in butyrate, *Z*-value 3.29). In contrast, the negative regulator of type I IFN responses, OASL1 (144) was down-regulated ≈1.5 fold (Supplementary Table 1k). The low magnitude of changes (Figure 6D), the counter-regulatory affects mediated by OASL1 and/or IRF2, and/or redundancy in the type I IFN system, may explain the lack of an effect on CHIKV loads.

## Discussion

To the best of our knowledge this paper represents the first study investigating the role of a high fiber diet and SCFAs on alphaviral rheumatic disease. Although a large body of work in non-infectious disease settings would argue that a high fiber diet should ameliorate inflammation, we illustrate herein that a high fiber diet modulated the CHIKV rheumatic immunopathology with increased edema, Th17/IL-17 activities, neutrophil infiltration, and psoriasis-like signatures, but reduced muscle damage. Although imbibing butyrate has also been shown to ameliorate inflammation outside the gut in multiple non-infectious disease settings (145–147), herein we show that butyrate consumption increased edema during CHIKV infection. Neither the high fiber diet nor drinking butyrate affected viral loads or anti-viral antibody responses; however, both clearly exacerbated CHIKV arthritic immunopathology.

The most dramatic change to CHIKV immunopathology mediated by a high fiber diet was the increase in edema and infiltrating neutrophils during peak arthritis. The high fiber diet mediated a range of changes to the CHIKV arthritic signature that supported neutrophil recruitment, survival, and activation (Table 1). Key drivers of neutrophil mediated inflammation are Th17 cell and IL-17, and although we have not formally demonstrated increased Th17 cells or IL-17 protein levels in the feet of CHIKV infected mice fed a high fiber diet, bioinformatic analyses suggest both are up-regulated in the high fiber group (Table 1, Supplementary Table 1a). Perhaps the most compelling bioinformatics support (for a fiber-mediated increase in pro-inflammatory and IL-17-mediated immunopathology after CHIKV infection) comes from the significant similarities between CHIKV arthritis in mice fed a high fiber diet and mouse models of inflammatory psoriasis (122) (Figure 4B). Although this was an unexpected and novel finding, there are salient parallels between CHIKV infection and psoriasis; (i) CHIKV after a high fiber diet and psoriasis both involve neutrophil recruitment (148), (ii) psoriasis can cause alopecia (149), and can have follicular involvement (124), with alopecia frequently reported by CHIKV patients (150–153) and also seen in some CHIKV mouse models (154, 155), (iii) although distinct from psoriatic lesions, skin manifestations (usually maculopapular rashes) are well-described for arthritogenic alphavirus infections in humans, but are rarely overt in mouse models, although they can be detected by IHC in certain settings (156), and (iv) perhaps most cogent, both psoriasis and CHIKV are associated with arthritis (157). The changes in skin immunopathology seen herein are consistent with the well-described ability of the gut microbiome to influence immunity in the skin via the so-called gut-skin-axis (158). A role for IL-17 in psoriasis has been established (159) and several studies in humans have also found an association between gut microbiota and Th17 responses (160, 161). Promotion of systemic Th17/IL-17 responses by acetate was also reported in another infectious disease setting where mice were infected orally with the gram-negative enteric bacteria *Citrobacter rodentium* (19). In addition, HDAC1 inhibition [an activity of butyrate (162)] has been shown to promote IL-17 transcription in human T cell lines *in vitro* (163). Whether a high fiber diet would predispose to more severe skin or rheumatic manifestations in humans after CHIKV infection remains to be determined, and any study seeing to establish a link would need to control for *inter alia* the multiple ways in which diet might affect arboviral infections (164).

The mechanisms whereby the high fiber diet would mediate the neutrophil-promoting changes during CHIKV arthritis are likely to be complex given the interaction of various SCFAs with multiple cell types (10, 18, 24, 66, 113), the infection of different cell types by CHIKV, and the complex interplay of anti-viral and inflammatory responses seen post infection (36, 41, 42, 51). Nevertheless, a likely key contributing mechanism highlighted herein is the down-modulation in the rM gene signature seen in feet during peak CHIKV arthritis in mice fed a high fiber diet (compared with peak CHIKV arthritis in mice fed a no fiber diet) (Figure 5). Adoption by infiltrating macrophages of a resolution phase phenotype is usually associated with loss of neutrophils and initiation of tissue repair (53, 54, 135). Both the presence of neutrophils and the GSEA argue that adoption of a rM phenotype is delayed in CHIKV arthropathy in mice fed a high fiber diet. A high fiber diet is believed generally to provide anti-inflammatory activities (10, 18) and anti-inflammatory drugs have been associated with delayed wound healing and/or tissue repair in a number of settings (165–167). Although a high fiber diet provided beneficial effects in influenza infections in mice, the mechanism also involved modulation of neutrophil-mediated tissue damage via the reshaping of macrophage functionality by SCFAs (66), perhaps arguing this is an important pathway whereby fiber mediates its effects on immunopathology.

Butyrate consumption increased edema during CHIKV arthritis, but the mechanisms involved appeared to be different from those seen for the high fiber diet. Bioinformatic analyses of RNA-Seq data suggested modulation of AP-1 and NF-κB activities and/or promotion of cell cycle arrest may be involved. The RNA-Seq and IHC analyses did not suggest any increases in pro-inflammatory cytokines (e.g., TNF, IL-1, and VEGF) or altered cellular infiltrates that might be associated with increased edema. Instead, we speculate that butyrate is promoting edema (at least in part) by acting on endothelial cells (24, 168). CHIKV infects endothelial cells *in vivo* (37, 169) and edema is a well-known symptom of CHIKV infection (47, 82). Endothelial barrier repair requires NF-κB activation (170, 171) and a NF-κB to AP-1 transition (172). Herein we provide evidence that butyrate promoted certain AP-1 activities and markedly inhibited canonical NF-κB pathways, consistent with previous studies on butyrate (31–34, 142). Such modulation of these key pathways may deoptimize endothelial barrier repair during CHIKV infection leading to increased edema. Cell cycle arrest may also play a role in promoting edema (both for butyrate Figure 6E and high fiber diet Figure 4C), as edema is a well-known side-effect of rapamycin (173, 174), a drug that also induces G1 arrest.

Perhaps curious is (i) the inability herein to recapitulate the effects of the high fiber diet with butyrate and (ii) the relatively minor effect butyrate had on transcription when compared with the high fiber diet (Figure 6D). For instance, butyrate has been widely reported to mediate G1 cell-cycle arrest via HDAC inhibition (125–127, 175). However, herein we see a more pronounced cell-cycle arrest signature in the high fiber group, as indicated by a widespread down-regulation of histone mRNAs (Supplementary Table 1h) that was not observed in the butyrate group (Supplementary Table 1k). The bioavailability of butyrate *in vivo* is actually quite low, so the circulating concentrations required for systemic HDAC inhibition may not be efficiently reached after drinking butyrate (175, 176). The more pronounced cell-cycle signature seen in the high fiber group may thus be due to additional or complimentary cell-cycle arrest activities mediated other SCFAs such as propionate and valerate (129–133).

There are a number of limitations for the studies described herein. Tissue levels of SCFAs were not assessed as part of this study; analyses which might provide some insights into the bioavailability issues discussed in the previous paragraph. RNA-Seq was also not performed on uninfected mice from the different dietary groups, limiting the interpretation of which changes in the transcriptome are caused by diet alone, and which are the result of the interplay of infection and diet. In addition, the different dietary regimens may mediate indirect effects on CHIKV arthritis, conceivably via changes in food/water uptake or altered physiology. We have also not addressed the question of whether a high fiber diet might change bone marrow neutrophil development or differentiation, or whether a high fiber diet affects circulating neutrophil numbers or differentiation states or the CHIKV infection-mediated transient leukopenia (169).

Arguably the most concerning aspect of CHIKV disease is the chronic arthralgia (joint pain), which can be protracted and is often difficult to manage with existing medications (36). We show herein that the significant differences in neutrophil infiltrates seen during peak arthritis (Figure 3C, Day 6.5), were not apparent during chronic arthritis [nominally deemed to be day 30 post infection in this model (55)] (Figure 3C, Day 30). Viral CPE and tissue damaging processes are largely over by the time the chronic phase of disease manifests (55), with the anti-inflammatory activity of fiber conceivably able to provide benefit during this later stage of CHIKV disease. Animal models of chronic arthralgia have not been established and so investigating the potential benefits of a high fiber diet for chronic alphaviral arthralgia would likely require human studies. Although chronic alphaviral arthralgia likely involves inflammatory pain, the arthralgia can have neuropathic characteristics (36), which are not usually considered to be effectively managed with anti-inflammatory interventions.

The observations made herein for acute CHIKV rheumatic immunopathology contrasts markedly with most reports in the field that describe disease amelioration by high fiber diets and SCFAs. However, the majority of such studies were conducted in non-infectious disease settings, with the role of fiber and SCFAs in infectious diseases largely unexplored, especially in scenarios where there is a robust systemic infection and widespread tissue damage, with a need for tissue repair. Some other studies have also shown deleterious effects from fiber and SCFAs (68, 177), reinforcing the notion that the health benefits of fiber and SCFAs may often be quite setting dependent.

## Data Availability Statement

The raw RNA-Seq data has been deposited to NCBI;BioProject ID, PRJNA555135; Submission ID, SUB5943019. SAMN12301865 to 7—High fiber diet, feet day 6.5 post CHIKV infection. SAMN12301868/69/70—No fiber diet, feet day 6.5 post CHIKV infection. SAMN12301871/2/3—Butyrate, no fiber diet, feet day 6.5 post CHIKV infection. SAMN12301874/5/6—Water, no fiber diet, feet day 6.5 post CHIKV infection. All gene sets are provided in Supplementary Table 1.

## Ethics Statement

All mouse work was conducted in accordance with the Australian code for the care and use of animals for scientific purposes as defined by the National Health and Medical Research Council of Australia. Mouse work was approved by the QIMR Berghofer Medical Research Institute animal ethics committee (P1060 A705603M) and was conducted in biosafety level-3 facility at the QIMR Berghofer. Mice were euthanized using carbon dioxide.

## Author Contributions

NP, BT, TTL, and JG: undertook the experiments and analyzed the data. TL: interpreted the histology. TH, TA, PM, YP, and HN: bioinformatics. EN and VL: methodology, interpretation, and manuscript review. NP and AS: funding acquisition. AS: conceptualized the study and wrote the manuscript.

### Conflict of Interest

The authors declare that the research was conducted in the absence of any commercial or financial relationships that could be construed as a potential conflict of interest.
