# Supplementary material for: Exacerbation of Chikungunya Virus Rheumatic Immunopathology by a High Fiber Diet and Butyrate
Source: Front Immunol. 2019 Nov 26;10:2736. doi: 10.3389/fimmu.2019.02736 (PMC6888101; doi:10.3389/fimmu.2019.02736)
Supplement: Supplementary file 1 [file Data_Sheet_1.PDF]

Diet **SF11-025**

Modified AIN93G Rodent Diet all CHO as Gel Crisp Starch

A semi-pure diet formulation for laboratory rats and mice based on AIN-93G.

- All CHO has been replaced with Gel Crisp starch.
- Gel Crisp is a modified high amylose starch made from maize starch

Calculated Nutritional Parameters	
Protein	19.40%
Total Fat	7.00%
Crude Fibre	4.70%
AD Fibre	4.70%
Digestible Energy	16.3 MJ / Kg
% Total calculated digestible energy from lipids	16.00%
% Total calculated digestible energy from protein	21.00%
% Total calculated digestible energy from carbohydrate	62.00%

Ingredients	
Casein (Acid)	200 g/Kg
Gel Crisp Starch	636 g/Kg
Canola Oil	70 g/Kg
Cellulose	50 g/Kg
DL Methionine	3.0 g/Kg
Calcium Carbonate	13.1 g/Kg
Sodium Chloride	2.6 g/Kg
AIN93 Trace Minerals	1.4 g/Kg
Potassium Citrate	2.5 g/Kg
Potassium Dihydrogen Phosphate	6.9 g/Kg
Potassium Sulphate	1.6 g/Kg
Choline Chloride (75%)	2.5 g/Kg
AIN93 Vitamins	10 g/Kg

Calculated Amino Acids	
Valine	1.30%
Leucine	1.80%
Isoleucine	0.90%
Threonine	0.80%
Methionine	0.80%
Cystine	0.06%
Lysine	1.50%
Phenylanine	1.00%
Tyrosine	1.00%
Histidine	0.60%
Tryptophan	0.30%

Calculated Total Minerals	
Calcium	0.47%
Phosphorous	0.32%
Magnesium	0.10%
Sodium	0.12%
Chloride	0.16%
Potassium	0.40%
Sulphur	0.23%
Iron	75 mg/Kg
Copper	7.3 mg/Kg
Iodine	0.2 mg/Kg
Manganese	18 mg/Kg
Cobalt	No data
Zinc	53 mg/Kg
Molybdenum	0.15 mg/Kg
Selenium	0.3 mg/Kg
Cadmium	No data
Chromium	1.0 mg/Kg
Fluoride	1.0 mg/Kg
Lithium	0.1 mg/Kg
Boron	2.1 mg/Kg
Nickel	0.5 mg/Kg
Vanadium	0.1 mg/Kg

Calculated Total Vitamins	
Vitamin A (Retinol)	4 000 IU/Kg
Vitamin D (Cholecalciferol)	1 000 IU/Kg
Vitamin E (a Tocopherol acetate)	78 mg/Kg
Vitamin K (Menadione)	1 mg/Kg
Vitamin C (Ascorbic acid)	None added
Vitamin B1 (Thiamine)	6.1 mg/Kg
Vitamin B2 (Riboflavin)	6.3 mg/Kg
Niacin (Nicotinic acid)	30 mg/Kg
Vitamin B6 (Pryridoxine)	7 mg/Kg
Pantothenic Acid	16.5 mg/Kg
Biotin	200 ug/Kg
Folic Acid	2 mg/Kg
Inositol	None added
Vitamin B12 (Cyancobalamin)	103 ug/Kg
Choline	1 470 mg/Kg

Calculated Fatty Acid Composition	
Myristic Acid 14:0	Trace
Palmitic Acid 16:0	0.30%
Stearic Acid 18:0	0.10%
Palmitoleic Acid 16:1	No data
Oleic Acid 18:1	3.90%
Gadoleic Acid 20:1	0.10%
Linoleic Acid 18:2 n6	1.50%
a Linolenic Acid 18:3 n3	0.98%
Arachadonic Acid 20:4 n6	No data
EPA 20:5 n3	No data
DHA 22:6 n3	No data
Total n3	0.98%
Total n6	1.51%
Total Mono Unsaturated Fats	3.98%
Total Polyunsaturated Fats	2.50%
Total Saturated Fats	0.50%

BSI JAS-ANZ FEEDSAFE ACCREDITED

Supplementary Figure 1.

High fiber diet formulation

Diet SF09-028	Modified AIN93G Rodent Diet, No Added Fibre, No Added Starch

A semi-pure diet formulation for laboratory rats and mice based on AIN-93G. This formulation satisfies the nutritional requirements for growth of rats and mice. Some modifications have been made to the original formulation to suit locally available raw materials.

- Fibre, starch and Dextrinised starch has been replaced by dextrose

Calculated Nutritional Parameters	
Protein	19.4%
Total Fat	7.0%
Crude Fibre	0.0%
AD Fibre	0.0%
Digestible Energy	16.9 MJ / Kg
% Total calculated digestible energy from lipids	16.0%
% Total calculated digestible energy from protein	21.0%

Ingredients	
Casein (Acid)	200 g/Kg
Dextrose Monohydrate	686 g/Kg
Canola Oil	70 g/Kg
DL Methionine	3.0 g/Kg
Calcium Carbonate	13.1 g/Kg
Sodium Chloride	2.6 g/Kg
AIN93 Trace Minerals	1.4 g/Kg
Potassium Citrate	2.5 g/Kg
Potassium Dihydrogen Phosphate	6.9 g/Kg
Potassium Sulphate	1.6 g/Kg
Choline Chloride (75%)	2.5 g/Kg
AIN93 Vitamins	10 g/Kg

Calculated Amino Acids	
Valine	1.30%
Leucine	1.80%
Isoleucine	0.90%
Threonine	0.80%
Methionine	0.80%
Cystine	0.06%
Lysine	1.50%
Phenylanine	1.00%
Tyrosine	1.00%
Histidine	0.60%
Tryptophan	0.30%

Calculated Total Minerals	
Calcium	0.47%
Phosphorous	0.32%
Magnesium	0.09%
Sodium	0.12%
Chloride	0.16%
Potassium	0.40%
Sulphur	0.23%
Iron	73 mg/Kg
Copper	7.1 mg/Kg
Iodine	0.2 mg/Kg
Manganese	18 mg/Kg
Cobalt	No data
Zinc	53 mg/Kg
Molybdenum	0.15 mg/Kg
Selenium	0.3 mg/Kg
Cadmium	No data
Chromium	1.0 mg/Kg
Fluoride	1.0 mg/Kg
Lithium	0.1 mg/Kg
Boron	3.3 mg/Kg
Nickel	0.5 mg/Kg
Vanadium	0.1 mg/Kg

Calculated Total Vitamins	
Vitamin A (Retinol)	4 000 IU/Kg
Vitamin D (Cholecalciferol)	1 000 IU/Kg
Vitamin E (a Tocopherol acetate)	78 mg/Kg
Vitamin K (Menadione)	1 mg/Kg
Vitamin C (Ascorbic acid)	None added
Vitamin B1 (Thiamine)	6.1 mg/Kg
Vitamin B2 (Riboflavin)	6.3 mg/Kg
Niacin (Nicotinic acid)	30 mg/Kg
Vitamin B6 (Pryridoxine)	7 mg/Kg
Pantothenic Acid	16.5 mg/Kg
Biotin	200 ug/Kg
Folic Acid	2 mg/Kg
Inositol	None added
Vitamin B12 (Cyancobalamin)	103 ug/Kg
Choline	1 470 mg/Kg

Calculated Fatty Acid Composition	
Myristic Acid 14:0	No data
Palmitic Acid 16:0	0.40%
Stearic Acid 18:0	0.10%
Palmitoleic Acid 16:1	No data
Oleic Acid 18:1	4.20%
Gadoleic Acid 20:1	0.10%
Linoleic Acid 18:2 n6	1.51%
a Linolenic Acid 18:3 n3	0.98%
Arachadonic Acid 20:4 n6	No data
EPA 20:5 n3	No data
DHA 22:6 n3	No data
Total n3	0.98%
Total n6	1.51%
Total Mono Unsaturated Fats	3.98%
Total Polyunsaturated Fats	2.50%
Total Saturated Fats	0.50%

BSI JAS-ANZ FEEDSAFE ACCREDITED

Supplementary Figure 2.

No fiber diet formulation

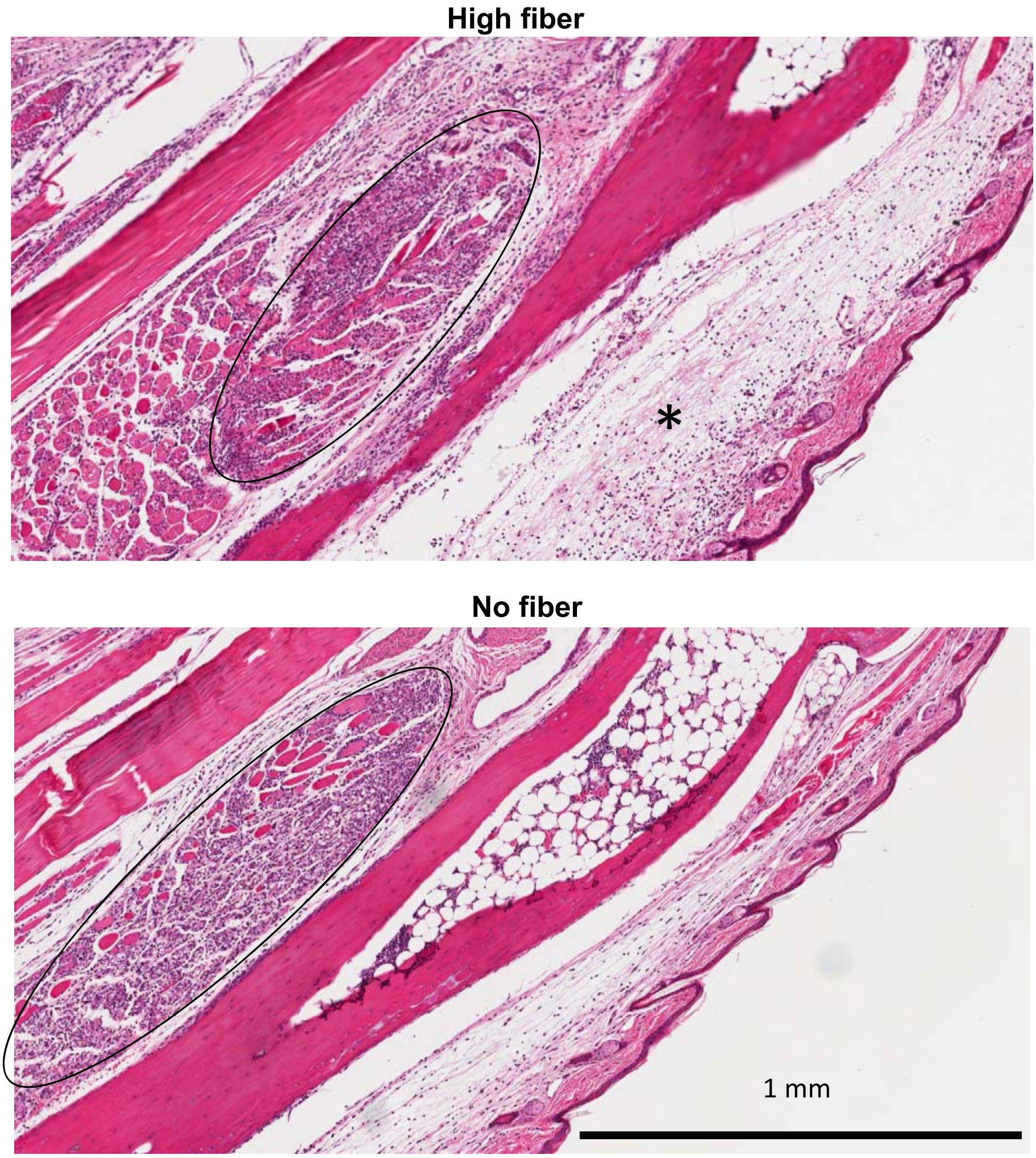

Supplementary Figure 3. H&E staining of feet 6.5 days post infection.

Mice fed a high fiber or a no fiber diet . * indicates edema. Black ovals indicate areas with high densities of leukocyte infiltrates.

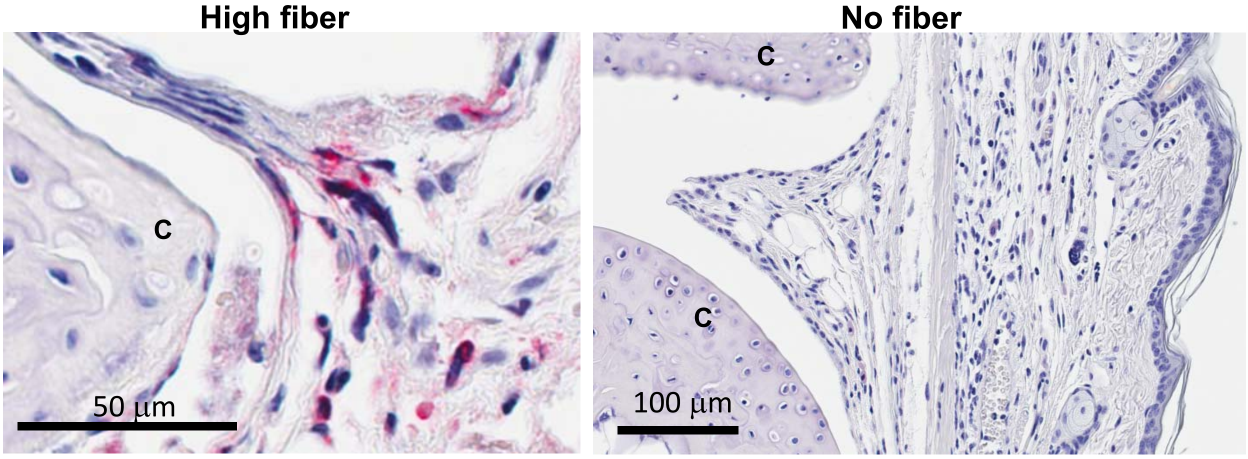

Supplementary Figure 4. Anti-Ly6G staining of synovia 6.5 days post infection.

Mice fed a high fiber or no fiber diet; red staining indicates neutrophils. C – joint cartilage.

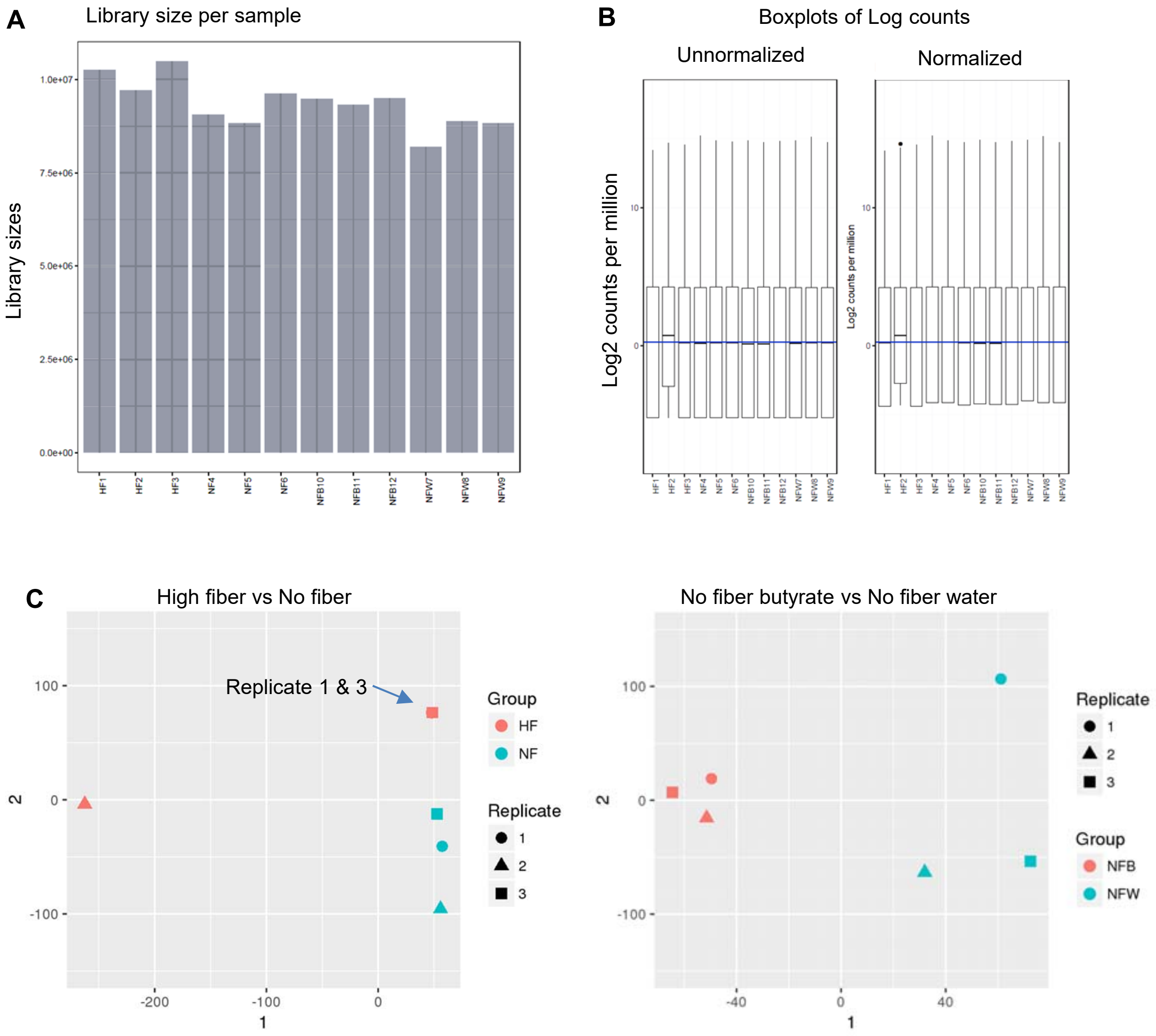

Supplementary Figure 5. RNA-Seq QC

(A) Library size of each sample. (B) Boxplots of Log counts. (C) MDS plots (MultiDimensional Scaling) after TMM normalized (Trimmed mean of M-values normalization Method), logCPM (counts per million) values.

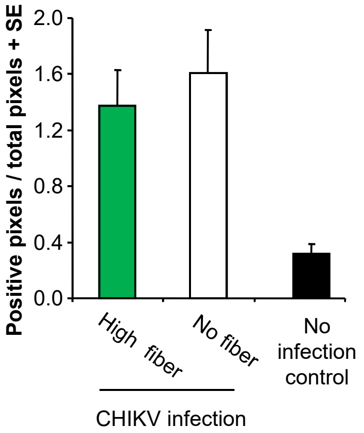

Supplementary Figure 6. IHC using anti-CD3 staining of feet 6.5 days post infection.

Mice fed a high fiber or a no fiber diet were infected with CHIKV and after 6.5 days feet were stained with anti-CD3 (A0452; Dako, North Sydney, Australia) with detection using Warp Red Chromogen Kit (Biocare Medical, Concord, CA, USA) and analysed by Aperio Pixel count. White pixels (e.g. edema) are not counted in the total pixel count. N=3/4 feet and mice per group.

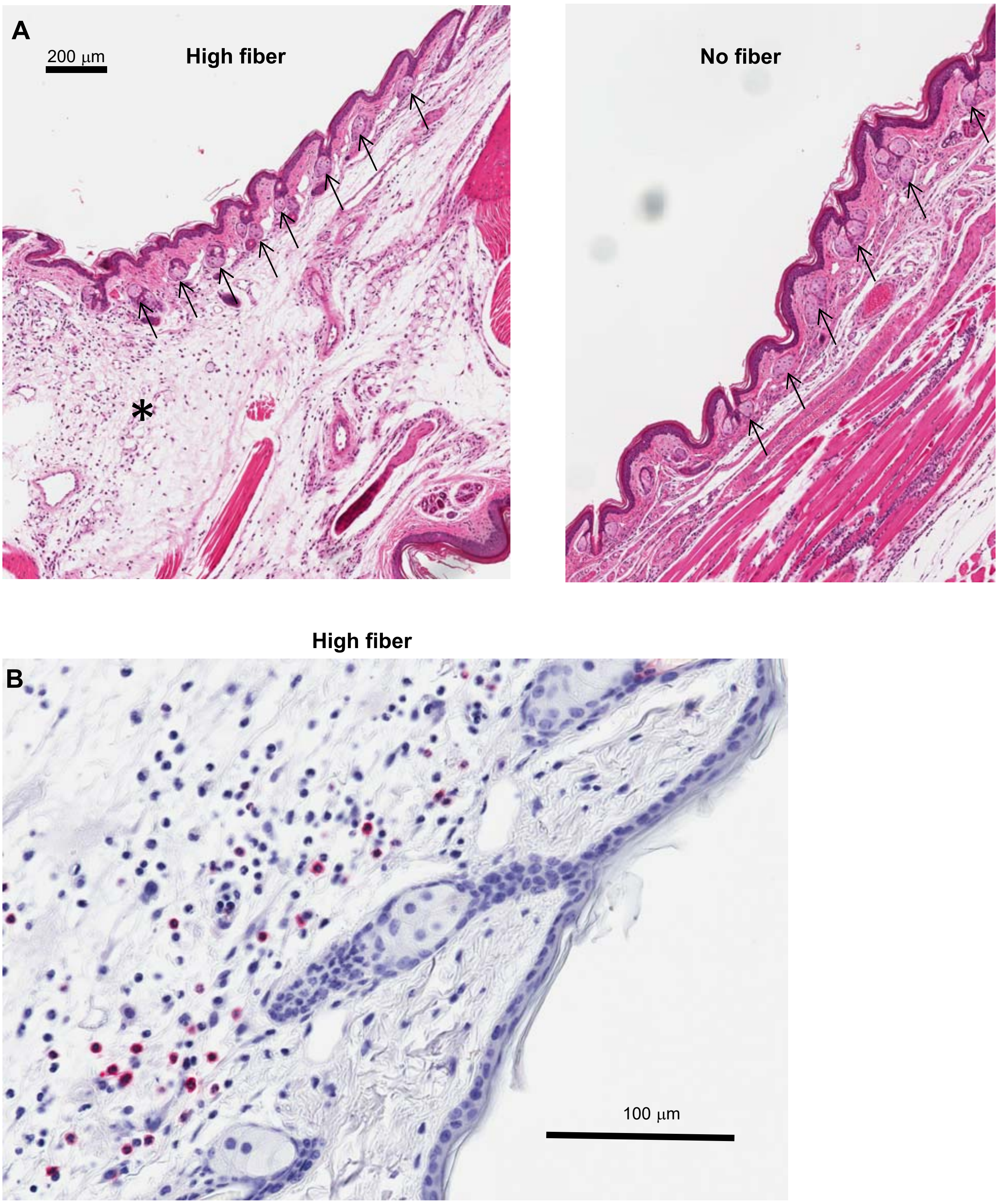

Supplementary Figure 7. H&E and IHC staining after high and no fiber diets

Mice fed a high fiber or a no fiber diet were infected with CHIKV and after 6.5 days feet were analyzed by H&E and IHC staining. (**A**) H&E staining. Arrows indicate sebaceous glands/hair follicles next to areas of edema. * indicates edema. (**B**) Anti-Ly6G staining (red).

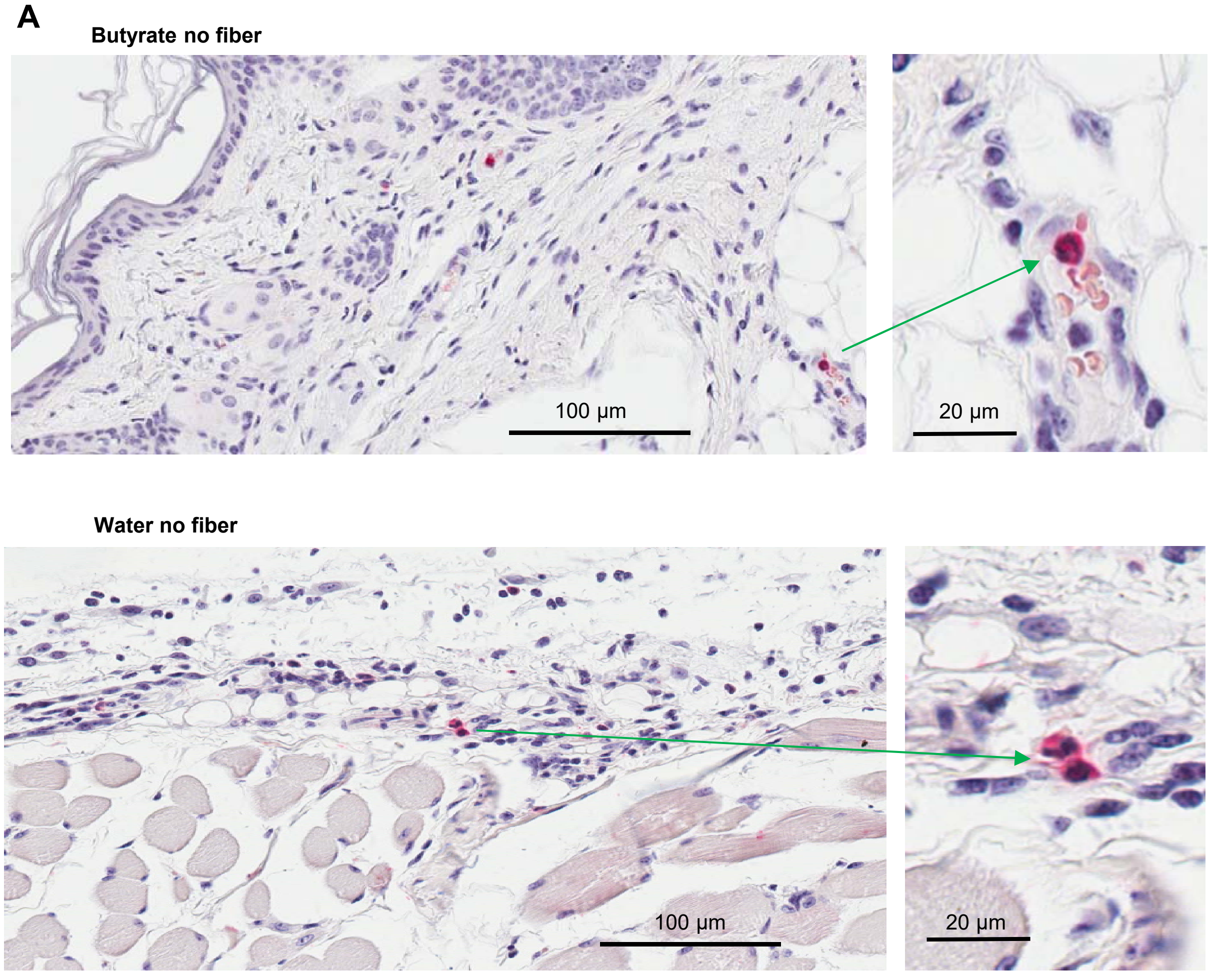

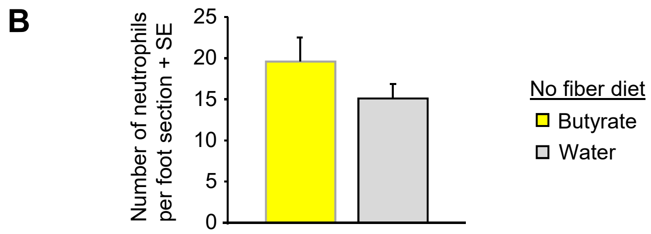

Supplementary Figure 8. Neutrophil staining for butyrate vs water

(**A**) Neutrophil staining (anti-Ly6G, red) for feet from mice drinking butyrate versus water (both no fiber) day 6.5 post CHIKV infection. Enlargements are shown (green arrows). (**B**) Aperio pixel count determinations of red staining had insufficient signal to noise ratios, given the low number of red staining neutrophils. Neutrophils were thus counted manually for 3/4 sections per foot and 6 feet and mice per group, providing neutrophil counts for n=20/21 anti-Ly6G-stained sections per group. Statistics by t test p=0.24.

A

Transcription factor	Z-value	Butyrate mediated change
Irf2_Irf1_Irf8_Irf9_Irf7	3.29	down[1]
Hcfc1_Six5_Smarcc2_Zfp143	2.50	up
Cebpe	2.30	up
Hey1_Myc_Mxi1	1.92	down
Foxj3_Tbl1xr1	1.79	up
Zic2	1.73	up
Tfap2c	1.64	down
Junb_Jund	1.63	down
Myf6	1.57	down
Tcf3	1.57	up
Sp1	1.51	down
Nrf1	1.49	up

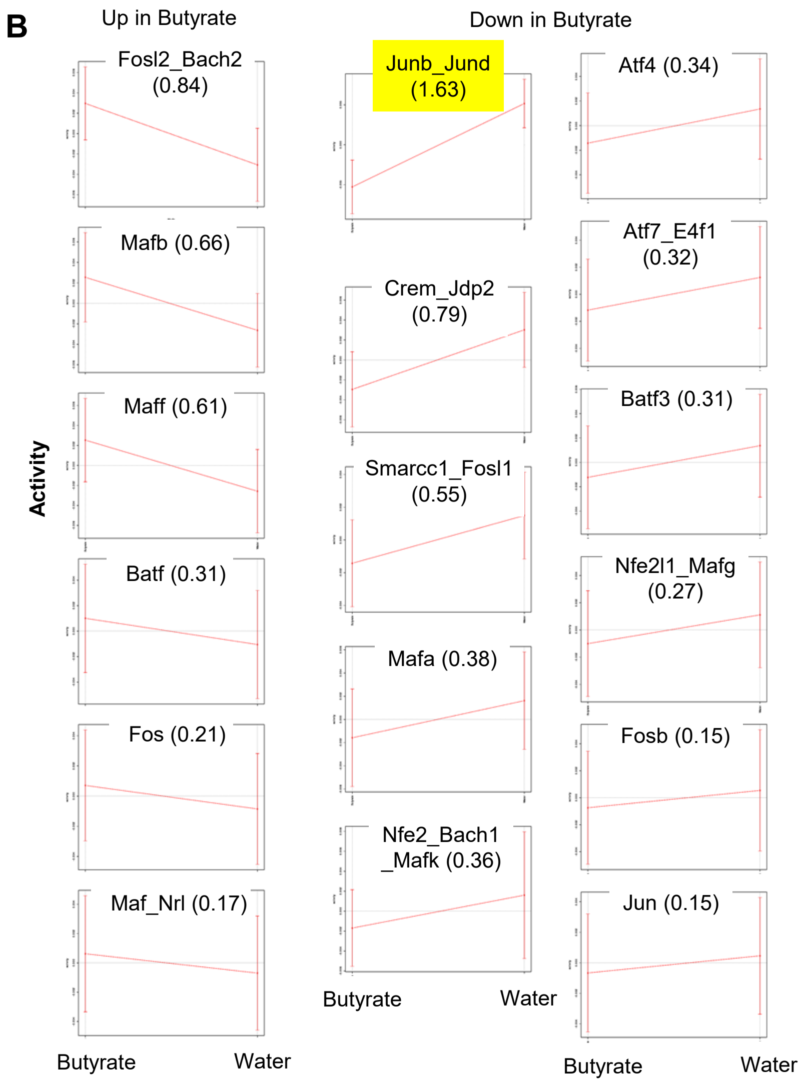

Supplementary Figure 9. ISMARA showing AP-1 family members

ISMARA analysis of the Buytrate vs Water RNA-Seq data. ISMARA quantifies significance as a Z-value, with a site with a higher Z value ranked as more significant than one with a lower Z value. The activity score is an arbitrary score provided by the program and if error bars overlap significance is deemed not to have been reached significance. Junb _Jund is deemed to have reached significance (yellow highlights). (**A**) Top transcription factor sites are shown. The full data set is provided in **Supplementary Table 1m**. [1] Note IRF2 is a repressor. (**B**) Activity graphs for AP-1 family member.

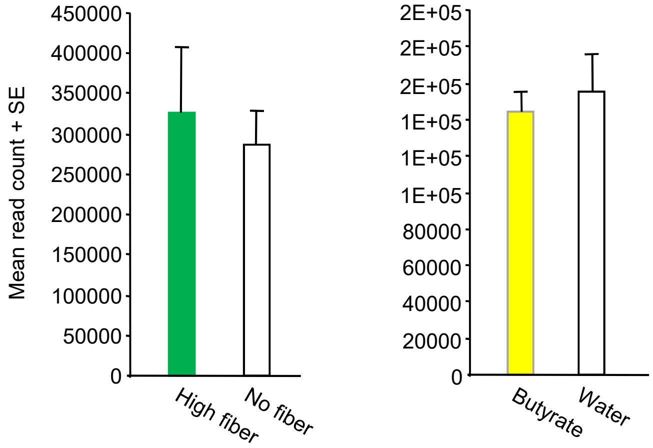

Supplementary Figure 10. RNA-Seq reads mapping to the CHIKV genome

RNA-Seq of feet of mice taken 6.5 days post infection with CHIKV also provided reads that can be mapped to the CHIKV genome. Reads were mapped to the CHIKV genome (KT449801) using STAR with Ensembl release v93 (after adapter trimming using cutadapt). Mean read counts are shown from n=3 RNA-Seq samples per group.
